# Long COVID: sustained and multiplied disadvantage

**DOI:** 10.5694/mja2.51435

**Published:** 2022-03-06

**Authors:** Evelyne de Leeuw, Aryati Yashadhana, Danielle Hitch

**Affiliations:** ^1^ Centre for Health Equity Training Research and Evaluation (CHETRE) USNW Sydney Sydney NSW; ^2^ Ingham Institute Sydney NSW; ^3^ CHETRE South Western Sydney Local Health District Sydney NSW; ^4^ Western Health Sunshine Hospital Melbourne VIC; ^5^ Deakin University Geelong VIC

**Keywords:** Public policy, COVID‐19, Social determinants of health, Socioeconomic status

The pandemic will be with us for a long time; we need to engage with its inequities

Policy and institutional preparedness for the coronavirus disease 2019 (COVID‐19) pandemic recovery is essential^1^ because the roots of the pandemic and its resolution are deeply systemic.^2^ The COVID‐19 pandemic disproportionately affects certain groups and populations. Generally, they are labelled as “vulnerable”, “marginalised” or “disadvantaged”, and these groups may be considered at risk from a medical perspective and/or from the perspective of their opportunities to function and participate in the community.^3^ But there is great and refined differentiation within these populations, whereby the varying waves have highlighted various inequitable and devastating effects. We followed recent guidance by Dahlgren and Whitehead^4^ for our understanding of differential effects of the COVID‐19 pandemic and one of its expressions (“long COVID”) across Australian populations, and the long term impacts on health, wellbeing and economic resilience. The classic Dahlgren and Whitehead “rainbow” representation of the layered and nested nature of determinants of health, and their causes does not necessarily account for unfair, avoidable, differential and systemic inequities in health. Dahlgren and Whitehead have recently demonstrated that the Diderichsen model^5^ offers additional sophistication to map cascading differential causes of cause of health inequity (Box). In fact, the combined frameworks have already been applied to understanding the unfair and differential consequences of COVID‐19 on minority groups.^6^ Lockdowns and other instances of TTIQ (test, trace, isolate and quarantine) and their enforcement have markedly different consequences for people in housing situations, for instance, which do not allow for spatial requirements; labour situations that do not allow reduced mobility; or social conventions that are destructive of family and kinship bonds. There is clearly a role for (primary) health care in addressing health inequities,^7^ including factful systems advocacy.

Box 1 **Long COVID health inequity heuristic framework**

**Figure:**
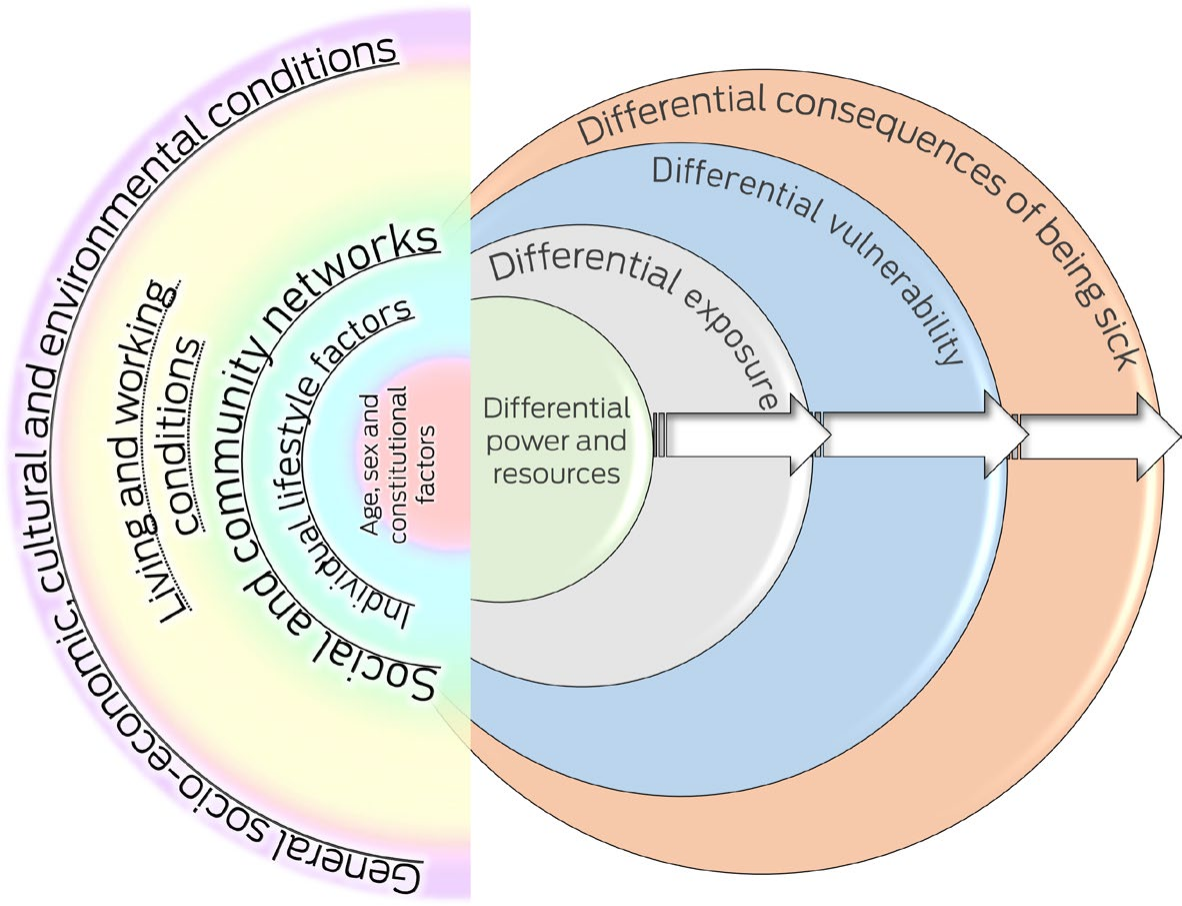
The Rainbow Model of Social Determinants of Health was adapted from Dahlgren and Whitehead^4^ and Diderichsen et al.^5^

Extant literature on the distribution and inequitable nature of the pandemic has mostly focused on infections, case numbers and vaccination rates. But scholars and commentators have increasingly identified and advocated critical fault lines in “glocal” (where global and local meet) society.^2^, ^8^ Public health and political leadership have taken the numbers and patterns to reassure affected localities (neighbourhoods, communities, local government areas, states and territories, the Commonwealth, and occasionally global levels) that there will be an end to the pandemic, and that there will be a degree of “new normal”. However, there is now growing awareness of the evidence gap regarding the impact of social determinants.^9^

The recognition of “living with COVID‐19” in a “new normal” only peripherally acknowledges continuing patterns of inequity and vulnerability, both in terms of acquiring an infection as well as maintaining immunity (either through vaccination or recovery). What is worse is the looming sustained wave of post‐COVID‐19 social and health impacts, which are also inequitably distributed. In short, the disadvantaged continuing to suffer more and longer. Precarious lives are further cast in uncertain futures.

In this article we identify the effect of long COVID across communities that were already struggling before the pandemic, which will suffer more from infection and will be devastated interminably from the ongoing cascade of social (eg, work and employment, housing and service access, disability support) and health (exacerbating chronic suffering and comorbid conditions) consequences.

## Long COVID

Soon after the initial waves of the pandemic had affected populations in OECD countries, such as Italy, the United Kingdom, Canada and the United States, there were reports that people who should have recovered from COVID‐19 were experiencing ongoing debilitating symptoms. These syndromes were colloquially labelled “long COVID”. The nature and precise aetiology of long COVID remains ill‐defined, and no diagnostic criteria currently exist. It has been suggested that hyper‐inflammation caused by long COVID links to neurodegeneration and cognitive decline.^10^ The multisystem impacts of long COVID are now becoming evident, with a recent systematic review identifying 55 distinct symptoms associated with this syndrome.^11^ Eighty per cent of patients experienced one or more symptoms after infection (14–110 days), including fatigue, headache and “brain fog”. Parallels with chronic fatigue and myalgic encephalomyelitis have been observed. On 6 October 2021, the World Health Organization published a consensus statement on the nomenclature and clinical dimension of long COVID and the amended terminology is now “post COVID‐19 condition”.^12^

From an equity perspective, the over‐representation of chronic conditions among disadvantaged (and often racialised) populations (eg, Indigenous Australians)^13^ increases the risk of both COVID‐19 acute severity and long COVID. People with long COVID have reported significant stigma, difficulties in accessing services and returning to full time work, trouble maintaining important relationships and life roles, and barriers to engaging in activities of daily living.^14^ Australian data confirm this.^15^ The infection risk for severe acute respiratory syndrome coronavirus 2 (SARS‐CoV‐2) is associated with age, immune status, and certain pre‐existing non‐communicable diseases such as obesity, asthma etc. One of the few predictive models available for long COVID has found associations with age, body mass index, female sex and the number of symptoms experienced within the first 7 days of infection.^16^ Each of these factors is already profoundly driven by the social determinants of health and health inequity.

The enduring effects of long COVID in groups that already experience disadvantage and inequality will make livelihoods more perilous. The detrimental health effects of what is called the “precariat” — “gig economy” workers (ride‐share drivers, food delivery riders, workers in precarious jobs, hosts of short term rentals etc), who are led to believe that they will achieve greater choice and more freedom in their livelihoods at the cost of appropriate social protections such as insurance, unemployment security, and old‐age investment — have been identified.^17^ Disadvantage stemming from these failures in the current (absence of the) social contract is only exacerbated by compounding barriers to living one’s life to the full.

Commentators have already identified that the pandemic has exposed the critical fault lines in society and between societies. But to our knowledge, no‐one has, as yet, warned for the longer term destabilising and incapacitating social, economic and health consequences of the next stage in the life of/with SARS‐CoV‐2.

## A research and action agenda

We have all witnessed, on a global scale, how devastating the pandemic has been. A literature review published in 2021^18^ is a helpful tool to assess the current economic and econometric tools that have been deployed to create images of the wellbeing economy impacts of COVID‐19. They also identify the inequitable labour, health, gender, discrimination and environmental developments that are a result of the initial disease waves. Those who were worse off across many determinants of health inequity will be even worse off after only partially recovering. This will be particularly true for populations with intersectionally determined comorbid conditions, such as socio‐economically disadvantaged groups, Indigenous communities, people with varying abilities (or disabilities) in residential care, vulnerable older people etc.

For a social justice‐driven recovery in a post‐COVID‐19 era (adopting the “building back better” mantra, perhaps),^19^ it is imperative that two things are taken on board by the partners in our social (health) contract at all levels and jurisdictions:

• “It ain’t over for some until it’s over for all.” Building back better needs to embrace the full spectrum of disease emergence, spread, control, follow‐up, and health care delivery in every stage of prevention, management and investment in care, explicitly including long COVID.
• Planning for this must involve not just rhetorical allusions to priority populations, as peddled by governments in late 2020 when repeatedly Indigenous Australians and aged care residents and carers were identified as such, without any meaningful operational action. Critical intersectionality creates pockets of deep and irreconcilable injustice and health inequity if public and non‐government organisations maintain a siloed mantra of priorities.

## Realising building back fairer

What is required to pursue and realise these aspirations?

Debates on the nomenclature and definition of long COVID must be communicated and implemented across health professionals and their institutions to enable consistent and adequate clinical diagnosis and management as well as appropriate social responses. This process must include people with lived experience for these criteria to be meaningful and relevant to the group to which they are referring. Availability of and access to data on long COVID that are disaggregated by socio‐economic status, geography, and ethnicity and cultural alignment will assist in building a foundational evidence base in which action research responses can be developed. The PROGRESS‐Plus protocol is a helpful tool to assemble and communicate the dimensions of disadvantage that will need to be addressed.^20^

However, to make a reliable and lasting impact on the prevention of entangled disadvantage, Australia should also focus on working in collaboration with equity‐seeking populations and communities most affected to understand lived realities and potential solutions. Policy and its development (or lack thereof) in response to long COVID is also key, as will be its integration into a nationally consistent and regionally administered patient‐centred health and social response.^21^ We must consider how structural inequities, such as housing availability and affordability, lack of adequate social protection, and marginalisation from health systems, may further entrench disadvantage in the face of long COVID.

Dealing with the fall‐out of the pandemic, on all fronts, will require the tangible commitment to deliver across sectors and for everyone.

## Competing interests

No relevant disclosures.

## Provenance

Not commissioned; externally peer reviewed.
